# Development of CRISPR/Cas9-mediated CD16b^-/-^ and CD32a^-/-^ promyelocytic cell lines to study FcγR signaling in human neutrophils

**DOI:** 10.3389/fimmu.2025.1633609

**Published:** 2025-10-02

**Authors:** José Antonio Cruz-Cárdenas, Alejandra López-Arredondo, Jorge Andrés Cázares-Preciado, Mabel Rodríguez-Gonzalez, Laura A. Palomares, Marion E. G. Brunck

**Affiliations:** ^1^ Escuela de Ingeniería y Ciencias, Tecnologico de Monterrey, Monterrey, Nuevo León, Mexico; ^2^ Laboratorio de Análisis de Moléculas y Medicamentos Biotecnológicos, Instituto de Biotecnología, Universidad Nacional Autónoma de México, Cuernavaca, Morelos, Mexico; ^3^ Instituto de Biotecnología, Universidad Nacional Autónoma de México, Cuernavaca, Morelos, Mexico; ^4^ Departamento de Inmunología, Instituto de Investigaciones Biomédicas, Universidad Nacional Autónoma de México, Ciudad de México, Mexico; ^5^ The Institute for Obesity Research, Tecnologico de Monterrey, Monterrey, Nuevo León, Mexico

**Keywords:** neutrophils, HL-60, FcγRIIIb, CD16B, FcγRIIa, CD32a

## Abstract

**Introduction:**

Neutrophils use Fc gamma receptors (FcγRs) to recognize IgG-opsonized pathogens, triggering antimicrobial functions including phagocytosis, ROS production, and cytokine release. CD16b, the most abundant FcγR on neutrophils, plays a key role in initiating these responses, while CD32a is another abundant FcγR on neutrophils that contributes to modulating immune functions. CD16b lacks an intracellular domain and its signaling mechanisms remain unclear. The prevalence of the CD16b-deficient phenotype on donor neutrophils is estimated at <1% of the global population, which complicates its study. To address this, we employed CRISPR/Cas9 to generate HL-60-derived neutrophil-like cells deficient for CD16b or CD32a, that facilitate investigation of their respective roles in neutrophil biology.

**Methods:**

We disrupted the *FCGR3B* or *FCGR2A* genes using CRISPR/Cas9 in the HL-60 cell line and differentiated clones into neutrophil-like cells using 1.3% DMSO. Functional assays were performed, including phagocytosis, ROS production, SYK phosphorylation, and cytokine responses.

**Results and discussion:**

Both CD16b^-/-^ and CD32a^-/-^ HL-60-derived clones maintained neutrophilic differentiation and phagocytic capacity but displayed impaired FcγR-mediated ROS production and SYK phosphorylation, with more pronounced defects in CD16b^-/-^ cells. Cytokine production was altered in both lines, with CD16b^-/-^ cells producing less IL-6 and IL-1β, and CD32a^-/-^ cells producing less TNF-α and IL-10. This model provides new insights into the distinct roles of CD16b and CD32a in neutrophil activation and immune responses.

## 1 Introduction

Neutrophils express Fc gamma receptors (FcγRs) that recognize and bind IgG-opsonized pathogens. Crosslinking of FcγRs activates neutrophils and prompts phagocytosis, reactive oxygen species (ROS) production, and cytokine production, ultimately resulting in pathogen elimination (1, 2). CD16b is the most abundant FcγR in neutrophil surfaces and is key to initiate phagocytosis and the production of ROS (3). Most insights into the function and signaling of CD16b have been obtained using selective antibody‐mediated crosslinking or enzymatic cleavage to remove surface-bound receptors (3–5). However, these strategies may induce non-specific activation through other receptors or by cytokine treatments to mobilize intracellular stores of the receptor (6). Selective crosslinking of CD16b signaling using agonists induces calcium mobilization and SYK phosphorylation, which culminate in ROS and cytokine production (4, 7, 8). Despite this critical role, the intracellular mechanisms through which CD16b mediates these functions remain elusive, as CD16b is a GPI-anchored receptor and lacks an intracellular domain. Membrane receptors that lack a cytoplasmic domain can still induce intracellular activation by coupling to additional adaptor molecules or other receptors that do contain signaling motifs (9). Earlier studies hypothesized that CD16b relies on co-engagement with signaling-competent receptors, including CD32a or complement receptor 3 (CR3) (CD11b/CD18) to mediate signaling events leading to ROS production (10, 11). However, there is no conclusive information about this potential co-engagement. Therefore, determining the extent of CD16b independent contribution to neutrophil responses remains challenging, highlighting the need for models that can dissect receptor-specific signaling pathways.

A handful of studies have reported a CD16b-deficient human neutrophil phenotype (12–15). We recently demonstrated that CD16b-deficient human neutrophils exhibit impaired phagocytosis and ROS production, which correlated with reduced actin polymerization and decreased SYK phosphorylation, supporting the importance of CD16b in triggering these functions (15). However, CD16b-deficient individuals have an estimated prevalence of <1% globally. Therefore, studying this phenotype using donor peripheral blood poses a significant challenge (13). The development of neutrophil models selectively lacking CD16b or other relevant FcγRs like CD32a could provide valuable tools to investigate the independent biological functions of these FcγRs. Here, we applied a CRISPR/Cas9 genetic editing strategy to produce HL-60-derived cell lines lacking either CD16b or CD32a. The surface FcγRs phenotype of DMSO-differentiated neutrophil-like cells showed potential regulations of the other FcγRs CD16a and CD64 in the absence of CD16b or CD32a. While phagocytosis efficiency was unaffected in the absence of CD16b or CD32a, compared to unedited HL-60, ROS production and SYK phosphorylation were significantly impaired in the knockout lines. Upon challenge with serum-opsonized *E. coli*, CD16b^-/-^ and CD32a^-/-^ neutrophil-like cells exhibited distinct cytokine response profiles, suggesting that FcγRs differentially modulate cytokine responses through different pathways. Altogether, this study presents novel cellular models as a valuable platform for dissecting the specific roles of CD16b and CD32a in neutrophil immune responses.

## 2 Methods

### 2.1 Cell culture

HL-60 cells (ATCC, cat. CCL-240) were cultured in IMDM (Gibco, cat. 12440046) supplemented with 20% fetal bovine serum (FBS, Gibco, cat. 26140079) and 1% of Antibiotic/Antimycotic (Gibco, cat. 15240062) at 37 °C in 5% CO_2_. Cells were passaged according to the manufacturer’s recommendations. Differentiation towards neutrophil-like cells was performed over 5 days by supplementing culture media with 1.3% DMSO (Sigma, cat. D2650). On day 3, cells were counted, and fresh media was added to maintain cell density <500,000 cells per mL. All experiments were completed before cell passage 30. All experiments were completed with passages <30.

### 2.2 Flow cytometry

Relevant receptors on cell surfaces were measured by flow cytometry. Briefly, 2 x10^5^ cells were incubated on ice with Fc block (Miltenyi Biotech, cat. 130-059-901) for 15 minutes to minimize non-specific staining, then stained with titrated concentrations of CD11b-AF700, CD15-BV786, CD64-BV650), CD32-APC, CD16b-PE and CD16A-AF405 (all details in **Supplementary Table S1**) for 30 min at 4 °C. Fixable viability stain 510 was added as a viability marker as per manufacturer’s instructions. The neutrophil-like phenotype was confirmed through the co-expression of CD11b and CD15 at day 5 of differentiation. After washing, samples were immediately acquired on a FACSCelesta fitted with 405nm, 488nm, and 640nm lasers, operated through the BD FACSDiva software v.8. Cytometer settings were validated as within the range of manufacturer’s recommendations using Cytometer Setup & Tracking (CS&T) beads (BD, cat. 642412) before each acquisition. Compensation controls were used at each acquisition using the CompBeads anti-mouse Ig, κ/Negative control compensation particle set (BD, cat. 552843) following the manufacturer’s recommendations. At least 20,000 compensated live singlets were acquired per sample. Data were analyzed using FlowJo v.10. Fluorescence-minus-one (FMO) controls were used to confirm gating. The gating strategy is presented in **Supplementary Figure S1**.

### 2.3 Gene editing using CRISPR/Cas9 mediated knock-out

**Figure 1:**
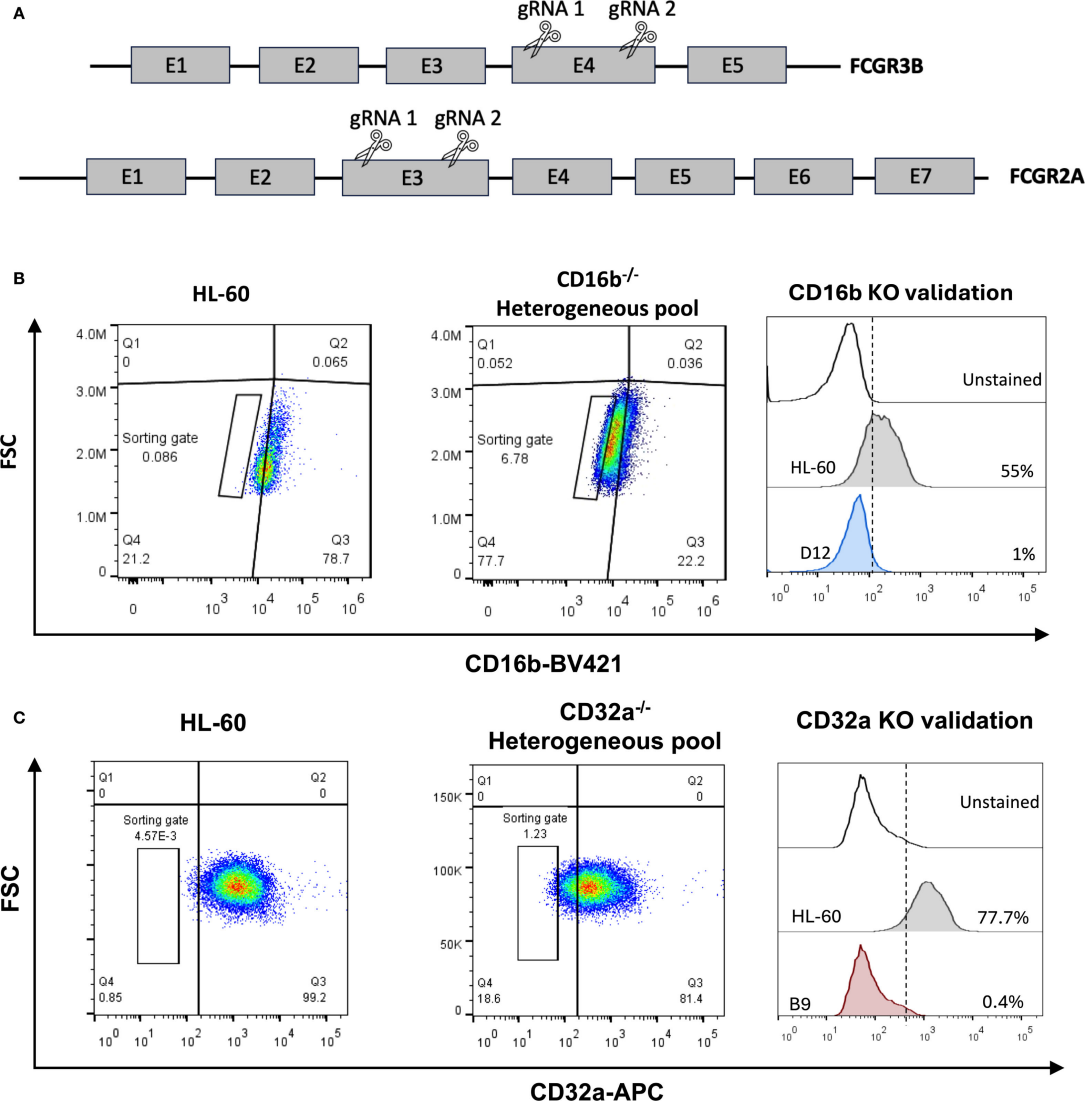
Generation of human neutrophil progenitor cell lines lacking CD16b or CD32a. **(A)** A 2-gRNA CRISPR-Cas9 strategy was employed to target CD16b (*FCGR3B* gene) and CD32a (*FCGR2A* gene) and abrogate protein expression through NHEJ. **(B)** Flow cytometry gating strategy employed to sort single cells deficient for CD16b surface expression, and to validate CD16b-KO clones. **(C)** Flow cytometry gating strategy employed to sort single cells deficient for CD32a surface expression, and to validate CD32a-KO clones. KO, knock-out.

A CRISPR-Cas9 strategy was designed to selectively disrupt the *FCGR3B* or *FCGR2A* genes in HL-60 cells. Two gRNAs per gene were designed to target conserved sequences on exon 4 or exon 3 of *FCGR3B* and *FCGR2A*, respectively (**Figure 1A**, **Supplementary Table S2**). The potential for off-targeting of each gRNA was analyzed using the CHOP-CHOP platform (16) and all predicted sites contained at least three mismatches relative to the original gRNA sequence, to limit potential off-target binding, although experimental validation of off-target effects was not performed. Guides were cloned individually in the lentiCRISPRv2 plasmid (Addgene, cat. 52961). Plasmids were transfected into HL-60 cells using the Neon transfection system (ThermoFisher Scientific). Briefly, 5x10^5^ HL-60 cells were electroporated with 1 pulse of 35 msec at 1350V. Twenty-four hours post-transfection, 1 μg/mL puromycin was added to the culture for 48 hours, followed by 0.5 μg/mL for 7 days. On day 10 post-transfection, single clones that were CD16b-deficient or CD32a-deficient were sorted into 96-well plates, using the antibody cocktail described in section 2.2. Cells were sorted using a Cytoflex SRT cell sorter fitted with 405 nm, 488 nm, 540 nm, and 630 nm lasers (Beckman Coulter), operated through the CytExpert software (v.2.3). Cell lines were validated by flow cytometry after 4 weeks of single-clones expansion on a BD FACSCelesta as described above.

### 2.4 Sanger sequencing

Genomic DNA of expanded clones was extracted using the kit QIAamp DNA Mini Kit (Qiagen, cat. 51306), following the manufacturer’s protocol. Primers were designed to flank the target regions (**Supplementary Table S2**). Target DNA was amplified by PCR and its sequence obtained using Sanger sequencing. Analysis of sequencing results was performed using Geneious Prime v2025.0.3 (Dotmatics). We compared the products of Sanger sequencing of the edited clones to sequences from unedited HL-60 cells, sequenced in parallel, and to sequences from the human reference sequences of CD16b (GenBank ID 2215) and CD32a (GenBank ID 2212).

### 2.5 Phagocytosis

Phagocytosis assays were performed using opsonized pHrodo *E. coli* bioparticles (Invitrogen, cat. P35361) following the manufacturer’s recommendations. Briefly, 20 μL of pHrodo *E. coli* bioparticles were opsonized with 20 μL of heat-inactivated human pooled sera (n=7) for 30 min at 37 °C and 100 rpm agitation in a 96-well plate. After opsonization, 1.75 x 10^5^ HL-60-differentiated neutrophil-like cells were added to wells and incubated for 30 min at 37 °C and 5% CO_2_ without agitation. At least 20,000 cells were then immediately acquired on a BD FACSCelesta flow cytometer as mentioned above. A phagocytic index was determined by dividing the mean fluorescence intensity (MFI) of bioparticle-treated cells by the MFI of untreated cells. This index reflects a relative quantity of phagocytosed particles per cell in the population, hence the average relative efficiency of phagocytosis at the cell level. The gating strategy is presented in **Supplementary Figure S2**.

### 2.6 Opsonized *E. coli* production

Opsonized *E. coli* were produced as previously described (15). Briefly, *E. coli* DH5-α cells were cultured in Luria-Bertani (LB) medium (Condalab, cat. 1551) overnight. After incubation, the cells were pelleted at 5,000 rcf, washed twice with a 10% glycerol solution, and aliquoted in 100 μL portions with a concentration 1.44 x 10^7^ CFU/mL. Culture aliquots were irradiated with UV light for 3 h and stored at -20 °C until use. Immediately before use, cells were washed with 1 mL of PBS at 16,000 rcf and resuspended in 20 μL of PBS. 20 μL of heat-inactivated human pooled sera (N = 7, samples from 3 healthy males and 4 healthy females) were added, and the mixture was incubated for 30 min at 37 °C with shaking at 100 rpm. The resulting opsonized-*E. coli* were washed with PBS before use. CFU/mL was determined by dilutions plating before irradiation. The ratio of opsonized *E. coli* to neutrophil used in all experiments was 7.2:1, meaning 7 bacteria per neutrophil.

### 2.7 ROS production

Production of ROS was evaluated by the oxidation of 1,2,3-dihydrorhodamine (DHR) (Invitrogen, cat. D23806). Briefly, 2x10^5^ cells/mL were incubated with 5μM of DHR and stimulated with either 100 ng/mL of phorbol 12-myristate 13-acetate (PMA, Sigma, cat. P8139) for 15 min at 37 °C or opsonized *E. coli* for 30 min at 37 °C. After incubation, the reaction was stopped by incubating the samples on ice for 10 min. Samples were washed and analyzed immediately on a BD FACSCelesta as described above, acquiring at least 20,000 single events. The gating strategy is presented in **Supplementary Figure S3**.

### 2.8 Cytokines

Cytokine production was induced by stimulating HL-60-differentiated neutrophil-like cells with opsonized *E. coli*. Briefly, 3x10^5^ cells were incubated with opsonized *E. coli* at 37 °C and 5% CO_2_ in 150 μL of complete media. Culture supernatants were collected after 8h incubation, and concentrations of proinflammatory cytokines were measured using a CBA human inflammatory cytokines kit (BD, cat. 551811) following the manufacturer’s recommendations. At least 5,000 bead-cytokines complexes were acquired on a BD FACSCelesta as per the manufacturer’s instructions. Data were analyzed using FCAP v2.0. Results are reported in pg/mL and all values below limit of detection (LOD) of 20 pg/mL were excluded from the analysis.

### 2.9 SYK phosphorylation

Phosphorylation of SYK kinase (pSYK) was measured in unstimulated HL-60-differentiated neutrophil-like cells and after stimulating with opsonized *E. coli*. Briefly, 2 x10^5^ cells were incubated with opsonized *E. coli* for 30 min at 37 °C and 5% CO_2_ without agitation. After incubation, the cells were washed in PBS and centrifuged at 300 rcf for 5 min at 4 °C. Cells were then fixed and permeabilized using the cytofix/cytoperm fixation/permeabilization kit (BD, cat. 554414), stained with anti-SYK (pY348)-PE (BD, cat. 558529) following the manufacturer’s protocols and immediately analyzed by flow cytometry on a BD FACSCelesta as described above. The stimulation index was calculated by dividing the pSYK MFI of stimulated samples by MFI of non-stimulated samples. The gating strategy is presented in **Supplementary Figure S4**.

### 2.10 Statistical analysis

Experiments are reported from at least 3 biological replicates. Data distributions were determined using Shapiro-Wilk tests. T-tests were used to compare 2 groups, and one-way ANOVA tests were used to compare >2 groups. Tukey’s *post-hoc* tests were applied to identify the groups with significant differences. All statistical tests were performed using Prism V.9 software (GraphPad). p-values <0.05 were considered significant.

## 3 Results

### 3.1 Production of the CD16b^-/^ *^-^* and CD32a^-/-^ HL-60 derived cell lines

We developed promyelocytic cellular models deficient for CD16b or CD32a. We used a CRISPR/Cas9 strategy with 2 guide RNA to induce double-strand DNA breaks, followed by non-homologous end joining (NHEJ) repair in the *FCGR3B* or *FCGR2A* genes (**Figure 1A**). Ten days after transfection and puromycin-based selection, edited cultures exhibited ∼78% of CD16b-negative cells and ∼17% of CD32a-negative cells for each model (**Figures 1B, C**). A gated population from the CD16b- and CD32a-negative cells was sorted, and after clonal expansion, we confirmed the absence of CD16b or CD32a in the clones using flow cytometry and Sanger sequencing (**Figures 1B, C**, **Supplementary Figures S5**, **S6**). In the expanded CD16b^-/-^ HL-60 clone, 2 mutations were identified in the *FCGR3B* gene, a substitution at G155C and the addition of a T at position 162 (**Supplementary Figure S5A**). In the expanded CD32a^-/-^ HL-60 clone, the *FCGR2A* gene harbored an A384C nonsense mutation (**Supplementary Figure S6A**), representative sanger sequencing chromatograms are present in **Supplementary Figure S6B**. In both cases, mutations also disrupted the open reading frame, resulting in premature stop codons (**Supplementary Figure S6C**).

### 3.2 HL-60 neutrophil-like cellular models downregulated CD11b upon opsonized *E. coli* stimulation

**Figure 2:**
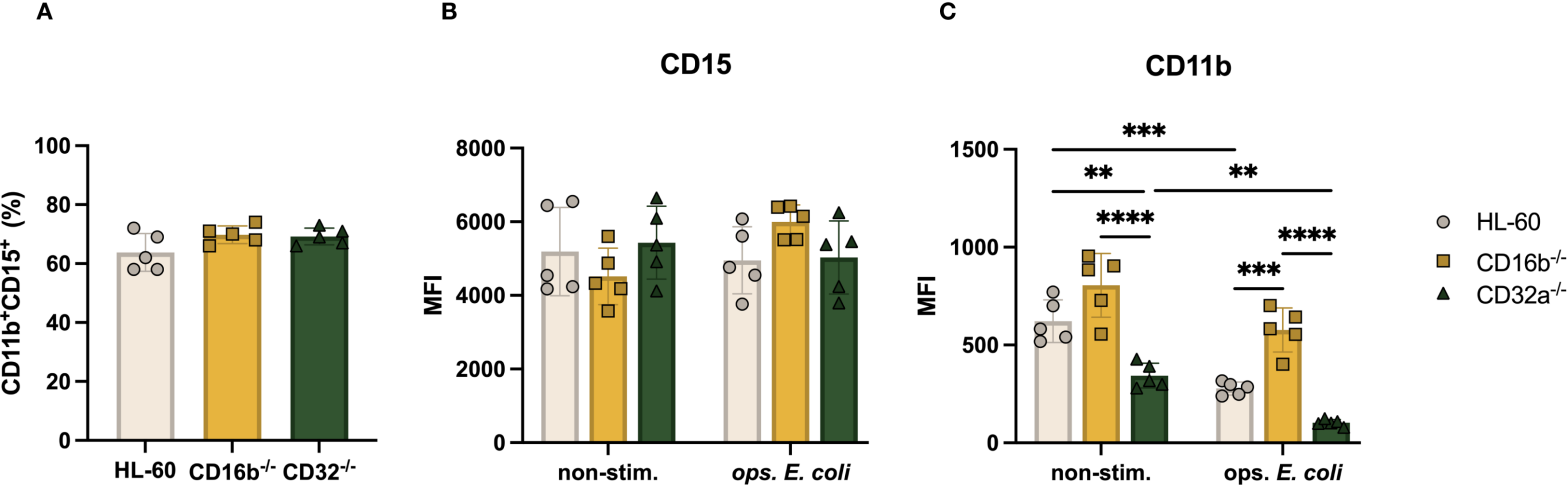
CD11b expression of CD16b^-/-^ and CD32a^-/-^ cells is impaired under non-stimulated and opsonized *E*. *coli* stimulation. **(A)** Proportion of CD11b^+^CD15^+^ cells, Median fluorescence intensity (MFI) of **(B)** CD15, **(C)** CD11b in the absence and opsonized *E*. *coli* stimulation. Gray circles: HL-60 neutrophil-like cells, yellow squares: CD16b^-/-^ cells, and green triangles: CD32a^-/-^ cells. non-stim: non-stimulated, ops. *E*. *coli*: stimulation with opsonized *E*. *coli*. N = 5 biological replicates for each group. ✱✱p<0.01, ✱✱✱p=0.0001 and ✱✱✱✱p<0.0001.

We characterized the surface phenotype of neutrophil-like cells obtained following a 5-day DMSO-induced differentiation. Overall, the proportions of CD15^+^CD11b^+^ cells in all cultures were similar, irrespective of genetic editing (average ~68%, **Figure 2A**). This suggested that the neutrophilic differentiation capacity of HL-60 cells was not affected by the selective knockout of the *FCGR3B* or *FCGR2A* genes. CD15 and CD11b are abundant receptors on neutrophil surfaces, and CD11b is regulated upon cell activation (17). We evaluated if the absence of CD16b or CD32a impacted the relative expression of CD15 or CD11b in steady- or activated states. The relative abundance of CD15 was similar on the surface of HL-60 and CD16b^-/-^ and CD32a^-/-^ both in non-stimulated and upon opsonized *E. coli* stimulation (**Figure 2B**). This suggested that CD16b or CD32a gene editing did not affect CD15 expression. In the absence of stimulation, the relative abundance of CD11b was similar between HL-60 cells and CD16b^-/-^ cells, consistent with earlier findings from a CD16b-deficient donor (15). In contrast, CD11b was significantly less abundant in the absence of CD32a, both in the absence of stimulation and following opsonized *E. coli* challenge (**Figure 2C**). Following opsonized *E. coli* stimulation, there was a reduced expression of CD11b in all 3 cell lines compared to their respective non-stimulated condition. This contrasts with previous findings from donor peripheral blood neutrophils where CD11b is upregulated following activation (18), suggesting HL-60 cells and derivatives do not fully recapitulate the robust CD11b mobilization on neutrophil surfaces measured on primary neutrophils following activation. This is also inconsistent with the upregulation of CD11b measured on the surface of CD16b-deficient neutrophils (15). Following opsonized *E. coli* challenge, CD11b expression was significantly higher in CD16b^-/-^ compared to CD32a^-/-^ or unedited HL-60 cells (**Figure 2C**). This could suggest that CD16b may play a role in CD11b regulation following opsonized *E. coli* stimulation through CD16b engagement (11).

### 3.3 CD16b^-/-^ but not CD32a^-/-^ neutrophil-like cells downregulated CD16a expression upon stimulation

**Figure 3:**
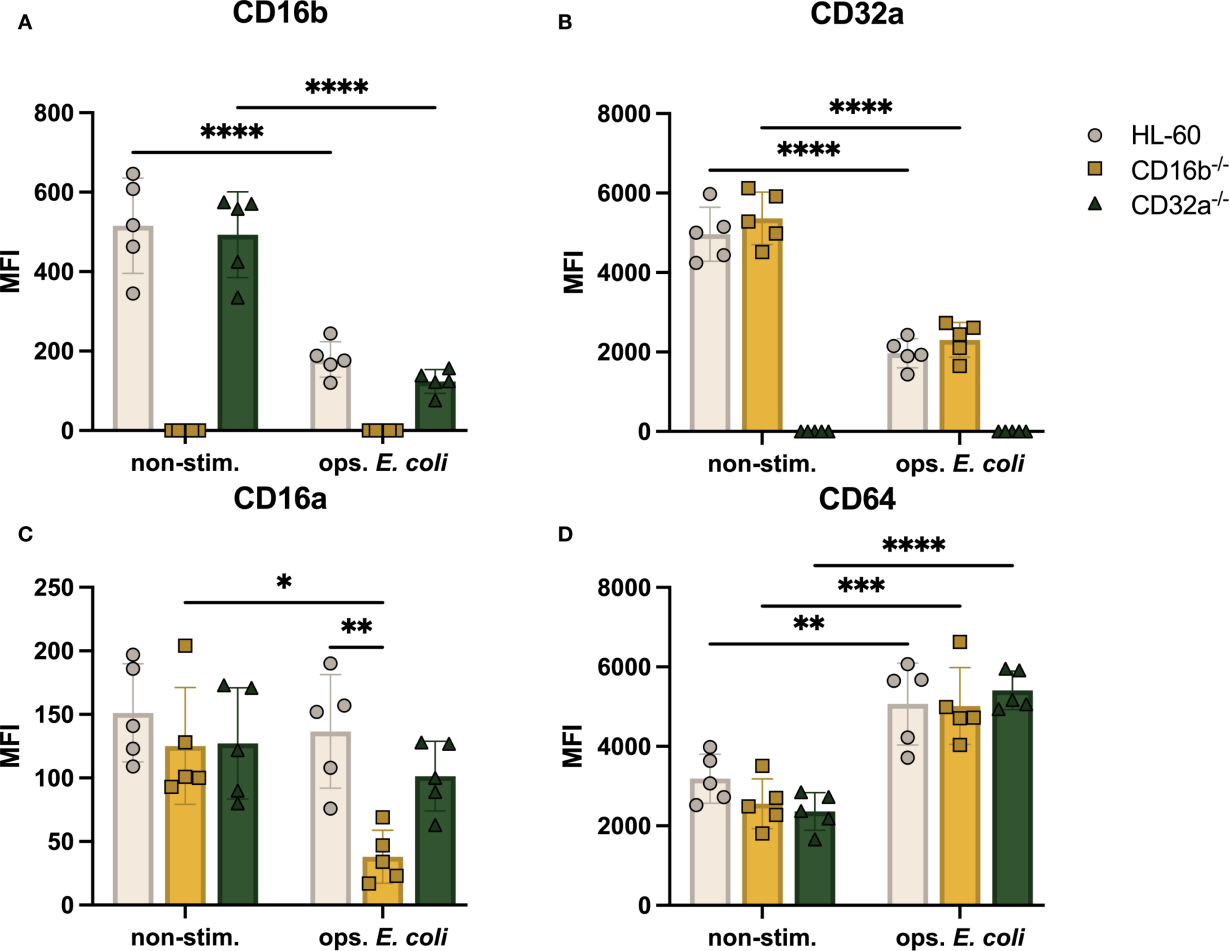
FcγRs are modulated on the surface of CD16b^-/-^ and CD32a^-/-^ neutrophil-like cell lines in non-stimulated state and following stimulation with opsonized *E*. *coli*. Median fluorescence intensity (MFI) of surface FcγR **(A)** CD16b, **(B)** CD32a, **(C)** CD16a, and **(D)** CD64. Gray circles: HL-60-derived neutrophil-like cells, yellow squares: CD16b^-/-^ neutrophil-like cells, and green triangles: CD32a^-/-^ neutrophil-like cells. non-stim: non-stimulated, ops. *E*. *coli*: stimulation with opsonized *E*. *coli*. N = 5 biological replicates performed on different days. Comparisons performed using one-way ANOVAs, ✱p<0.05, ✱✱p<0.01, ✱✱✱p=0.0001 and ✱✱✱✱p<0.0001.

Having identified the regulation of CD11b together with the genetic edition of CD16b, we wondered if other significant neutrophil receptors, such as FcγRs may be regulated. We found that the relative abundance of CD16b was similar in HL-60 and CD32a^-/-^ cells (**Figure 3A**), suggesting that the expression of CD16b may be regulated independently of CD32a. Upon opsonized *E. coli* challenge, both HL-60 and CD32a^-/-^ neutrophil-like cells downregulated CD16b expression, possibly due to receptor internalization or enzymatic cleavage, as proposed earlier (5, 15, 19). The expression of CD32a was also similar in HL-60 and CD16b^-/-^ cells in the absence of stimulation (**Figure 3B**), also suggesting independent regulation of both main FcγRs. Following opsonized *E. coli* challenge, CD32a expression was significantly downregulated in both HL-60 and CD16b^-/-^ neutrophil-like cells (p-value < 0.0001 for both, **Figure 3B**), likely evidencing the internalization of CD32a in phagolysosomes, in agreement with an early report (15).

The relative abundance of CD16a was similar in the 3 cell lines (**Figure 3C**) in the absence of stimulation. However, following opsonized *E. coli* stimulation, the expression of CD16a was significantly reduced in CD16b^-/-^ neutrophil-like cells (p-value = 0.015) but not in unedited and CD32a^-/-^ cells (**Figure 3C**). This is consistent with an earlier report where we have shown that the relative abundance of CD16a was reduced upon opsonized *E. coli* challenge in CD16b-deficient donor peripheral blood neutrophils but not in CD16b-expressing donor neutrophils (15). The current results are therefore consistent with a naturally occurring phenotype. We have proposed that in the absence of CD16b, CD16a may play a role of opsonized particles internalization during phagocytosis, causing the observed decreased abundance on the cell surface.

CD64 is rapidly upregulated in the context of bacterial infections or sepsis (20). This is consistent with an earlier study showing that, unlike other FcγRs, CD64 is upregulated following opsonized *E. coli* challenge. In this work, CD64 was significantly higher in CD16b-deficient peripheral blood neutrophils (15). Here, we found that CD64 expression was similar irrespective of the presence of CD16b or CD32a on neutrophil-like cells in the absence of stimulation and was significantly upregulated in all 3 cell lines following opsonized *E. coli* challenge (**Figure 3D**). A previous study showed that IFN-γ-induced CD64 does not mediate phagocytosis of IgG-opsonized beads in human neutrophils (3). This may suggest that CD64 is not involved in mediating phagocytosis or at least is not internalized in phagolysosomes.

### 3.4 CD16b^-/-^ and CD32a^-/-^ neutrophil-like cells maintained phagocytosis capacity but presented impaired ROS production

**Figure 4:**
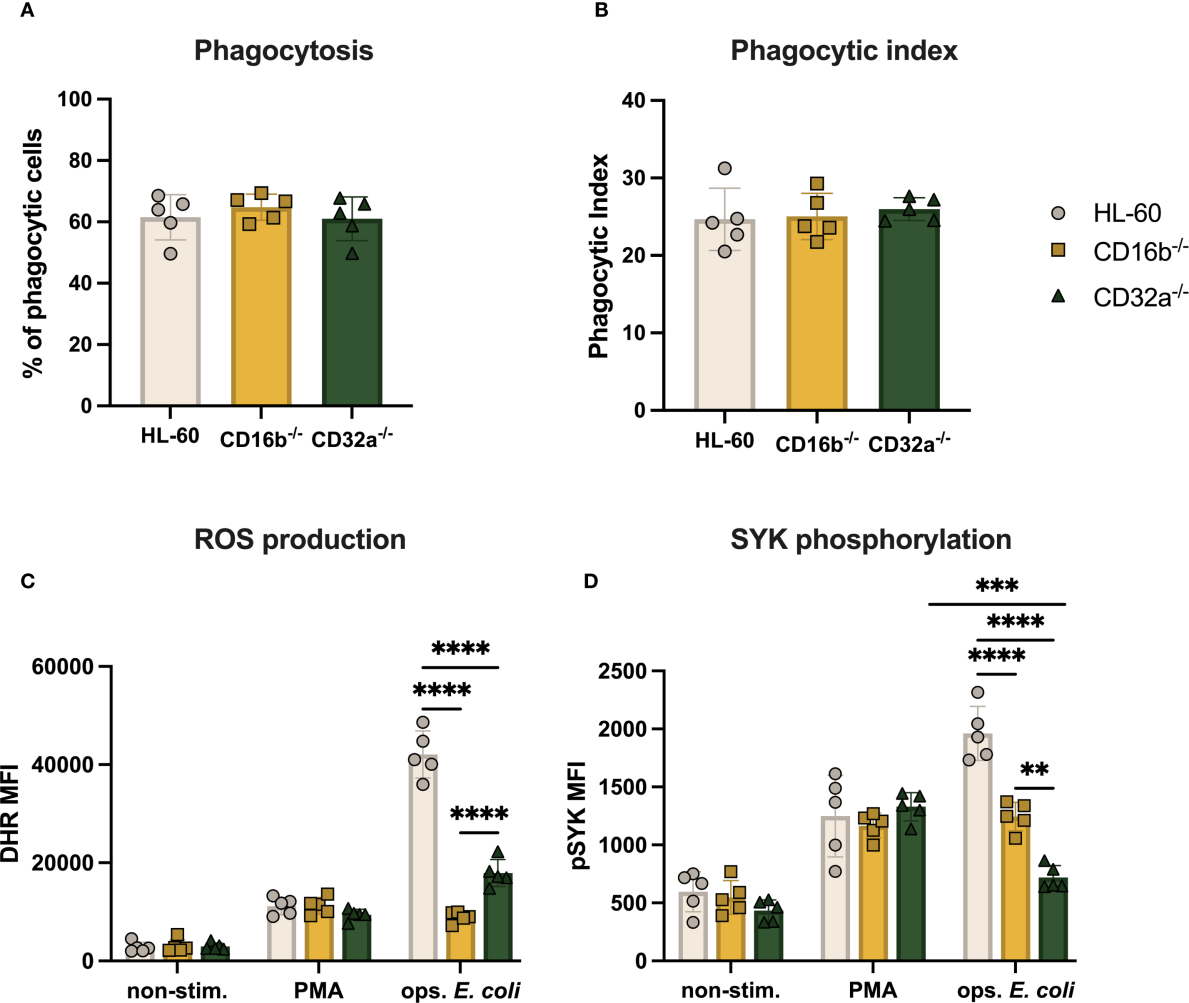
ROS production and SYK phosphorylation of CD16b^-/-^ and CD32a^-/-^ cells are impaired. **(A)** Percentage of phagocytosing cells per sample, **(B)** phagocytic index, **(C)** ROS production, and **(D)** Median fluorescence intensity (MFI) of pSYK following PMA (100 ng/mL) and opsonized *E*. *coli* stimulation. Gray circles: HL-60-derived neutrophil-like cells, yellow squares: CD16b^-/-^ neutrophil-like cells, and green triangles: CD32a^-/-^ neutrophil-like cells. non-stim: non-stimulated, ops. *E*. *coli*: stimulation with opsonized *E*. *coli*. N = 5 biological replicates performed on different days. Comparisons performed using one-way ANOVAs, ✱✱p<0.01, ✱✱✱p=0.0001 and ✱✱✱✱p<0.0001.

CD16b-deficient peripheral blood neutrophils have impaired phagocytic capacity (15). We investigated if this finding was replicated in edited neutrophil-like cells lacking CD16b and investigated whether CD32a deficiency affected key neutrophil functions in this model. Over half of all cultures (∼62%) exhibited phagocytosis following challenge with opsonized pHrodo *E. coli* bioparticles (**Figure 4A**). The phagocytic index was also similar, suggesting comparable mean phagocytic capacity per cell (**Figure 4B**). These results suggest that, unlike primary CD16b-deficient neutrophils (15), the absence of CD16b or CD32a in HL-60-derived neutrophil-like cells does not impair the phagocytic function. This discrepancy underscores potential limitations of the HL-60 model in replicating the functional behavior of primary neutrophils.

Previous studies have shown that CD16b and CD32a crosslinking promote ROS production (4). We also recently described that PMA stimulation resulted in similar levels of ROS irrespective of the presence of CD16b on peripheral blood neutrophil surfaces (15). Here, we similarly found that ROS production was comparable across the 3 cell lines following PMA stimulation (**Figure 4C**). This suggests that the PKC signaling pathway was not affected by the genetic editing performed in this work. In contrast, stimulation with opsonized *E. coli* led to significantly decreased ROS production in both CD16b^-/-^ and CD32a^-/-^ cells compared to HL-60 cells (p-value < 0.0001 for both, **Figure 4C**). Therefore, the current models replicate findings from peripheral blood neutrophils on CD16b and CD32a crosslinking and ROS production (4, 15). CD16b^-/-^ neutrophil-like cells produced significantly less ROS than CD32a^-/-^ cells upon opsonized *E. coli* stimulation (p-value < 0.0001), suggesting that CD16b crosslinking may be critically more involved in ROS responses following opsonized *E. coli* challenge, in line with a previous study (4).

### 3.5 CD16b^-/-^ and CD32a^-/-^ cell lines exhibited reduced SYK phosphorylation

SYK phosphorylation is involved in downstream signaling following FcγRs crosslinking, and is key in initiating ROS production (4, 7). We therefore investigated if the lower ROS produced by CD16b^-/-^ and CD32a^-/-^ cells resulted from altered pSYK upon FcγRs crosslinking. We found similar levels of pSYK in the 3 cultures in the absence of stimulation (**Figure 4D**). Following PMA stimulation, pSYK significantly and similarly increased in all 3 cell lines, suggesting stable PKC-mediated pathways (**Figure 4D**). In contrast, following opsonized *E. coli* stimulation, both CD16b^-/-^ and CD32a^-/-^ cultures exhibited significantly less pSYK compared to HL-60 cells (p-values < 0.0001, **Figure 4D**), supporting the key involvement of these receptors to trigger the production of ROS. CD16b^-/-^ neutrophil-like cells exhibited significantly higher pSYK compared to their CD32a^-/-^ counterpart (p-value < 0.0001, **Figure 4D**). While opsonized *E. coli* stimulation of HL-60 cells increased pSYK levels compared to PMA stimulation, CD16b^-/-^ cells exhibited similar pSYK irrespective of stimulation. This is in line with an earlier study showing that inhibiting SYK following CD16b blocking with an antagonist resulted in reduced ROS production, evidencing the role of CD16b in initiating the SYK signaling pathway (4). This also parallels previous findings of reduced pSYK following opsonized *E. coli* challenge in CD16b-deficient peripheral blood neutrophils (15).

Interestingly, CD32a^-/-^ cells exhibited similar levels of pSYK both in the absence of stimulation and upon opsonized *E. coli* challenge, while pSYK was significantly increased following PMA stimulation. This suggests that CD16b crosslinking cannot compensate for the absence of CD32a, to induce pSYK. This proposes a potential cooperative role of CD32a with CD16b in the activation of the SYK. Additionally, this suggests that the production of ROS following opsonized *E. coli* stimulation in CD32a^-/-^ cells was independent of SYK activation, possibly mediated by direct recognition of bacterial PAMPs protruding from opsonized *E. coli* as previously reported (15). The elevated pSYK levels observed following PMA stimulation could be due to a ROS-dependent feedback mechanism, whereby PMA-induced activation of PKC promotes NADPH oxidase-driven H_2_O_2_ production, which in turn enhances pSYK through redox-based mechanisms (21).

### 3.6 CD16b and CD32a deficiency altered cytokine production in neutrophil-like cells

Human peripheral blood neutrophils produce IL-8 in non-stimulated conditions (22), and others have shown that HL-60-derived neutrophil-like cells also spontaneously produced IL-6 and IL-8 (23). Here, the 3 cell lines also produced IL-8 in the absence of stimulation (**Supplementary Figure S7**). Interestingly, IL-8 concentrations were significantly lower in CD32a^-/-^ cells compared to CD16b^-/-^ and HL-60 cells (p-value = 0.0004 and 0.0009, respectively, **Supplementary Figure S7A**). In line with previous findings, IL-6 was also produced in the absence of stimulation by all 3 cell lines, but its concentration was significantly less in both CD16b^-/-^ and CD32a^-/-^ compared to unedited HL-60 cells (**Supplementary Figure S7B**). These findings suggest that the observed cytokine production may be influenced by oxidative stress associated with DMSO-induced differentiation (24), potentially creating a low-grade inflammatory state that primes the cells for basal cytokine release even in the absence of external stimulation.

**Figure 5:**
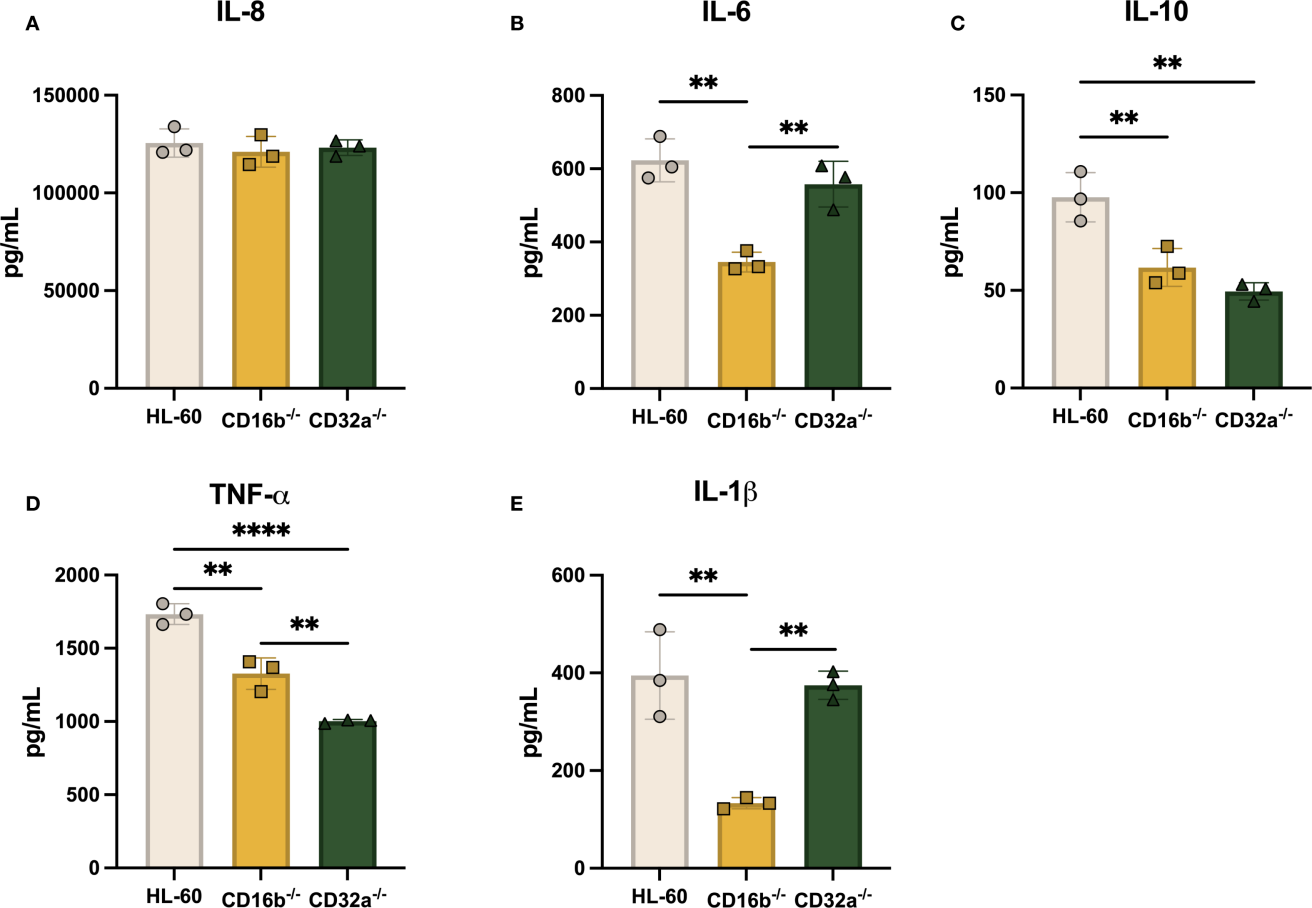
The production of proinflammatory cytokines following opsonized *E. coli* challenge is dependent on CD16b and CD32a. Concentration of **(A)** IL-8, **(B)** IL-6, **(C)** IL-10, **(D)** TNF-α, and **(E)** IL-1β in supernatants 8 h following opsonized *E*. *coli* stimulation. Gray circles: HL-60-derived neutrophil-like cells, yellow squares: CD16b^-/-^ neutrophil-like cells, and green triangles: CD32a^-/-^ neutrophil-like cells. N = 3 biological replicates performed on different days. Comparisons performed using one-way ANOVAs, ✱✱p<0.01 and ✱✱✱✱p<0.0001.

FcγRs crosslinking leads to the production of IL-8, IL-6, TNF-α, and IL-10 by human neutrophils (25). We measured IL-8, IL-6, IL-10, IL-1β, and TNF-α, in culture supernatants 8 h post-opsonized *E. coli* stimulation, as others have reported that maximum concentrations of cytokines are reached at this time (26). IL-8 concentrations were significantly higher compared to concentrations from non-stimulated cultures for the 3 cell lines (**Figure 5A**). Although IL-8 concentrations from CD32a^-/-^ cells were significantly lower than HL-60 and CD16b^-/-^ in the absence of stimulation (**Supplementary Figure S4**), upon opsonized *E. coli* challenge, IL-8 concentrations were similar across cultures.

A previous study has shown that IL-6 is produced after FcγRs crosslinking (27). IL-6 concentrations were significantly increased upon opsonized *E. coli* challenge in the 3 cell lines. Others have reported that IL-6 is produced in similar levels following selective CD16b or CD32a antagonistic inhibition in human peripheral blood neutrophils (25). Here, contrasting results were found, where CD16b^-/-^ cells exhibited reduced IL-6 production compared to CD32a^-/-^ and HL-60 cells (**Figure 5B**). This suggests that IL-6 production may rely more on CD16b crosslinking, supporting distinct roles of FcγRs in prompting cytokine production following opsonized *E. coli* stimulation.

IL-10 was measured in the 3 cell lines supernatants following opsonized *E. coli* challenge. However, both CD16b^-/-^ and CD32a^-/-^ cells produced similar concentrations of IL-10, which were significantly lower compared to concentrations measured in HL-60 cells (p-value = 0.0087 and 0.002, respectively, **Figure 5C**). TNF-α has similarly lower concentrations in both CD16b^-/-^ and CD32a^-/-^ cells compared to HL-60 cells (p-value = 0.0013 and < 0.0001, respectively). CD32a^-/-^ cells produced significantly less TNF-α compared to CD16b^-/-^ cells (**Figure 5D**). These findings indicate that both CD16b and CD32a contribute to the regulation of cytokines following receptor crosslinking. Moreover, the data suggest that CD32a crosslinking more efficiently activates downstream signaling pathways, leading to stronger induction of TNF-α production compared to CD16b (28, 29). IL-1β production was significantly reduced in opsonized *E. coli* stimulated CD16b^-/-^ cells compared to HL-60 and CD32a^-/-^ cells (**Figure 5E**). This suggests that CD16b crosslinking regulates IL-1β production. In summary, both CD16b^-/-^ and CD32a^-/-^ cell lines produced reduced levels of IL-10, CD16b^-/-^ cells produced less IL-6 and IL-1β upon opsonized *E. coli* stimulation, and CD32a^-/-^ cells produced less TNF-α. Altogether, this evidence suggests that FcγRs are key regulators of cytokine production (30).

## 4 Discussion

Dissecting FcγRs individual contributions using receptor-specific knockout models allows an unlimited source of cells to investigate their signaling and functional roles in human neutrophil functions. Here, we engineered HL-60-derived cellular models selectively lacking CD16b or CD32a to investigate the relevance of these FcγRs in relevant neutrophil antimicrobial responses. To the best of our knowledge, this is the first *in vitro* model to facilitate the study of FcγRs. Our gene editing strategy successfully introduced mutations in both genes, resulting in premature stop codons and disruption of the open reading frame. The *FCGR3B* gene contains a highly polymorphic exon 3 (31), and previous studies have reported exon 2 mutations as the cause of CD16b deficiency in some donors (15). However, to avoid variability due to polymorphisms, we targeted conserved regions in exon 4. This allowed for efficiency and consistent gene disruption across different *FCGR3B* variants.

A previous study reported that approximately 65% of HL-60 cells differentiated with 1.3% DMSO for 5 days co-expressed CD11b and CD15, and that extending differentiation to 7 days increased this proportion to about 80%. We observed similar co-expression percentages at day 5 (68%) and day 7 (77%) of differentiation (32). As FcγR relative expressions were higher at day 5 compared to day 7, we decided day 5 would be an optimal end point for functional assays. The absence of CD16b or CD32a did not affect neutrophil-like cell phagocytosis capacity, suggesting functional redundancy where other receptors may compensate for the absence in mediating phagocytosis (12). A recent report described decreased phagocytosis in CD16b-deficient neutrophils, which is contradictory to the present findings. This discrepancy may reflect differences between primary neutrophils and the HL-60 model in terms of functional and regulatory mechanisms. While the HL-60 model is valuable for dissecting individual receptor functions, its lack of phagocytosis impairment observed in primary CD16b-deficient neutrophils likely reflects compensatory mechanisms or intrinsic limitations of the model, potentially caused by distinct maturation states of the cells. The reduced ROS production observed in CD16b^-/-^ and CD32a^-/-^ cells following opsonized *E. coli* challenge suggests a key role of these FcγRs in triggering ROS production, consistent with previous studies (4, 15). In addition, we found that CD16b^-/-^ cells produced significantly less ROS than CD32a^-/-^ HL-60 cells. This is in line with previous studies showing that CD16b crosslinking induced higher levels of ROS production than CD32a crosslinking using selective agonists (4) and with data from CD16b^null^ neutrophils, which showed reduced ROS production upon opsonized *E. coli* stimulation (15).

Previous reports have shown downregulation of FcγRs after crosslinking (5, 15). CD16a is a high-affinity receptor on neutrophil surfaces and contributes to phagocytosis and cell activation (12). CD16a basal expression was higher in CD16b-deficient donor neutrophils compared to CD16b-expressing donor neutrophils and was decreased in CD16b-deficient donor neutrophils upon opsonized *E. coli* challenge, suggesting its contribution to phagocytosis (15). Here, we observed similar CD16a in the 3 cell lines in the absence of stimulation, and its downregulation in CD16b^-/-^ only, after opsonized *E. coli* challenge. This suggests a potential role of CD16a in phagocytosis of IgG-opsonized *E. coli*, also in agreement with previous studies (12, 15). However, further studies are needed to mechanistically evidence the role of CD16a in phagocytosis, and we propose that the presented model will support such research.

Others have reported that inhibition of SYK following FcγRs crosslinking resulted in decreased ROS production (4). We observed decreased pSYK in both CD16b^-/-^ and CD32a^-/-^ HL-60 upon opsonized *E. coli* challenge compared to unedited HL-60, similar to the behavior observed in CD16b-deficient primary neutrophils (15). Interestingly, the observed lower levels of pSYK in CD32a^-/-^ upon opsonized *E. coli* challenge suggest that CD16b may need to cooperate with CD32a to activate the SYK-mediated signaling pathway.

Here, baseline production of IL-8 was lower in CD32a^-/-^ cells but was restored to the levels of the other cell lines following opsonized *E. coli* stimulation. This may suggest that CD32a contributes to basal IL-8 production while induced IL-8 production may be driven in majority by CD32a-independent signals. This possible regulation requires significant additional research. Others have shown that selective CD16b crosslinking led to higher production of IL-10 compared to CD32a crosslinking (25). Our findings showed that the lack of either CD16b or CD32a resulted in reduced IL-10 production compared to HL-60. This suggests that both CD16b and CD32a crosslinking are needed to induce IL-10. On the other hand, reduced IL-6 and IL-1β concentrations were measured in CD16b^-/-^ cells only. This may suggest a more prominent role for CD16b in cytokine induction within this cellular system. Decreased TNF-α were measured following IgG-opsonized *E. coli* stimulation in CD16-deficient mice neutrophils (33). Furthermore, selective CD16b crosslinking leads to an increased TNF-α production compared to CD32a crosslinking (25). Here we reported a reduced capacity to produce TNF-α upon opsonized *E. coli* stimulation in both CD16b^-/-^ and CD32a^-/-^ compared to unedited cells, which may suggest a significant role of both these receptors in inducing TNF-α responses. In addition, we observed lower TNF-α concentrations in CD32a^-/-^ neutrophil-like cells compared to CD16b^-/-^, while the absence of CD16b more significantly impacted IL-6 and IL-1β production. This may suggest divergent downstream signaling pathways from these two FcγR. Future experiments using pathways-specific inhibitors should help dissect these signaling contributions in detail. DMSO differentiation intensifies oxidative stress (34), inducing a metabolic rewiring that may lead to a persistent proinflammatory state. This may explain cytokines measured in unstimulated cells, but also suggests that DMSO-differentiated cells which could have been activated by peptidoglycan and LPS from opsonized *E. coli* through upregulation of TLR 2/4 (35). Here we described FcγRs-mediated regulations of neutrophil responses in an *in vitro* model. DMSO-induced differentiation may lead to some immature cells in culture (36). Consequently, this model may not fully recapitulate all functional aspects of peripheral blood neutrophils. CD11b upregulation is a feature of activated peripheral blood neutrophils (15, 37). Here, we measured downregulation of CD11b upon opsonized *E. coli* challenge in CD32a^-/-^ and HL-60 but not in CD16b^-/-^ cells, suggesting that CD16b may be a regulator of CD11b mobilization (10).

FcγR plays a pivotal role in neutrophil antimicrobial responses. To the best of our knowledge, this is the first description of a neutrophilic cellular model genetically deficient for CD16b or CD32a. We observed impaired immune function, and a potential regulatory mechanism associated with the absence of these receptors. These differences may stem from cell line-specific regulations or may suggest non-redundant contributions of CD16b and CD32a to FcγR-mediated responses, including cytokine response and ROS production. Further research is required to fully elucidate the specific roles of individual FcγRs, and we propose this model may facilitate such studies. Finally, the HL-60 model, though valuable, presents limitations. DMSO-induced differentiation does not produce a fully mature phenotype. DMSO itself promotes oxidative stress, potentially impacting neutrophil functions (34, 36). In addition, as primary neutrophils are sensitive to manipulations and cannot be genetically edited. However, we have identified CD16b^null^ donors, whose neutrophils recapitulate features from the dHL-60 CD16b^-/-^ cell line, including CD16a internalization during phagocytosis, CD64 upregulation following opsonized bacteria challenge decreased ROS production and SYK phosphorylation patterns (15). Therefore, the developed model cell lines effectively recapitulated some FcγRs-specific features from primary neutrophils also lacking CD16b and could provide a platform for mechanistic studies of FcγR-mediated human neutrophil activation.

## Data Availability

The raw data supporting the conclusions of this article will be made available by the authors, without undue reservation.

## Supplementary material

Supplementary Table 1 Flow cytometry staining used for phenotype characterization.

Supplementary Table 2 Sequences of gRNAs for KO generation and primers for KO validations.

Supplementary Figure 1 Gating strategy for CD11b vs. CD15 phenotyping in neutrophil-like cells. FSC, forward scatter; SSC, side scatter.

Supplementary Figure 2 Gating strategy of phagocytosis experiments. FSC, forward scatter; SSC, side scatter. Unstimulated cells (gray), no opsonized pHrodo *E. coli* bioparticles. Stimulated cells (red), cells stimulated with opsonized pHrodo *E. coli* bioparticles. APC, Oxidized pHrodo.

Supplementary Figure 3 Gating strategy for ROS production (DHR oxidation) analysis by flow cytometry. Unstimulated (gray): DHR only, PMA-stimulated cells (100 ng/mL) (green), and opsonized *E. coli*-stimulated cells (red). FSC, forward scatter; SSC, side scatter.

Supplementary Figure 4 Gating strategy for SYK phosphorylation analysis by flow cytometry. Unstimulated (gray), PMA-stimulated cells (green), and opsonized *E. coli*-stimulated cells (red). FSC, forward scatter; SSC, side scatter.

Supplementary Figure 5 Mutations on *FCGR3B* gene from edited HL-60 caused frameshift and premature stop codons. **(A)** Alignment of *the FCGR3B* gene from HL-60 and CD16b^-/-^ HL-60 edited cells. Red boxes represent gRNA sequences, highlighted bases indicate mutation sites and dash lines (-) indicate gaps, representing base deletions leading to frameshifts. **(B)** Open reading frame (ORF) analysis of CD16b^-/-^ HL-60. Black asterisks indicate premature stop codons introduced by the genetic modifications. **(C)** Representative chromatograms of gRNA target sequence in unedited HL-60 and CD16b^-/-^ HL-60. Green boxes represent base substitutions and yellow boxes indicate indels.

Supplementary Figure 6 Mutations on *FCGR2A* gene from edited HL-60 caused frameshift and premature stop codons. **(A)** Alignment of *the FCGR2A* gene from HL-60 and CD16b^-/-^ HL-60 edited cells. Red boxes represent gRNA sequences, highlighted bases indicate mutation sites and dash lines (-) indicate gaps, representing base deletions leading to frameshifts. **(B)** Open reading frame (ORF) analysis of CD32a^-/-^ HL-60. Black asterisks indicate premature stop codons introduced by the genetic modifications. **(C)** Representative chromatograms of gRNA target sequence in unedited HL-60 and CD16b^-/-^ HL-60. Green boxes represent base substitutions and yellow boxes indicate indels.

Supplementary Figure 7 Constitutive IL-6 and IL-8 release by neutrophil-like cells depending on FcγR expression. **(A)** Concentration of IL-8 and **(B)** concentration of IL-6 in supernatants after 8 h of standard culture. Gray circles: HL-60-derived neutrophil-like cells, yellow squares: CD16b^-/-^ neutrophil-like cells, and green triangles: CD32a^-/-^ neutrophil-like cells. N = 3 biological replicates performed on different days. Comparisons performed using one-way ANOVAs, ✱p<0.05 ✱✱p<0.01, ✱✱✱p=0.0001.
