# Supplementary figures and images for: Development of CRISPR/Cas9-mediated CD16b-/- and CD32a-/- promyelocytic cell lines to study FcγR signaling in human neutrophils
Source: Front Immunol. 2025 Oct 2;16:1633609. doi: 10.3389/fimmu.2025.1633609 (PMC12528219; doi:10.3389/fimmu.2025.1633609)

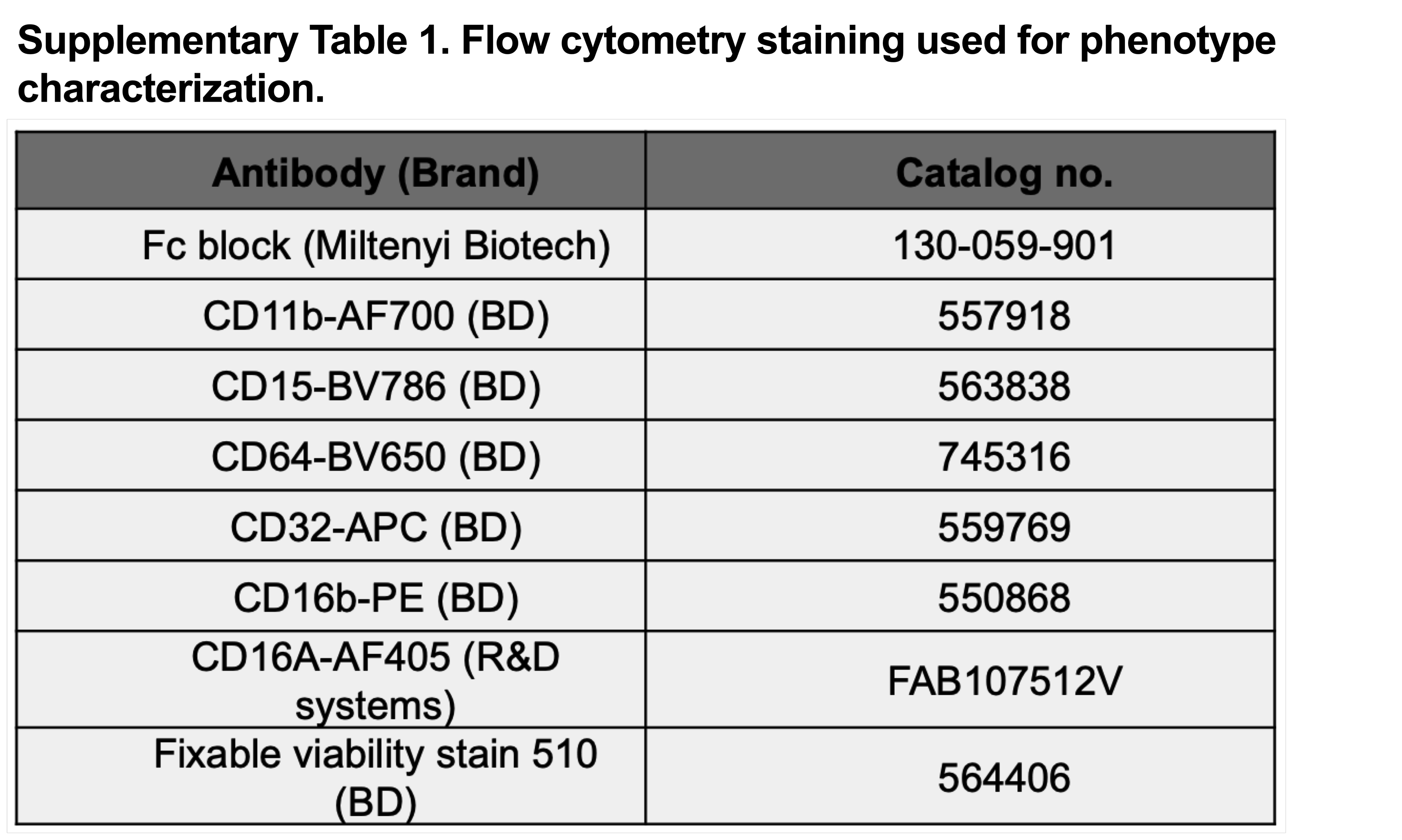

Supplement: Supplementary Table 1 — Flow cytometry staining used for phenotype characterization. [file Image8.tiff]

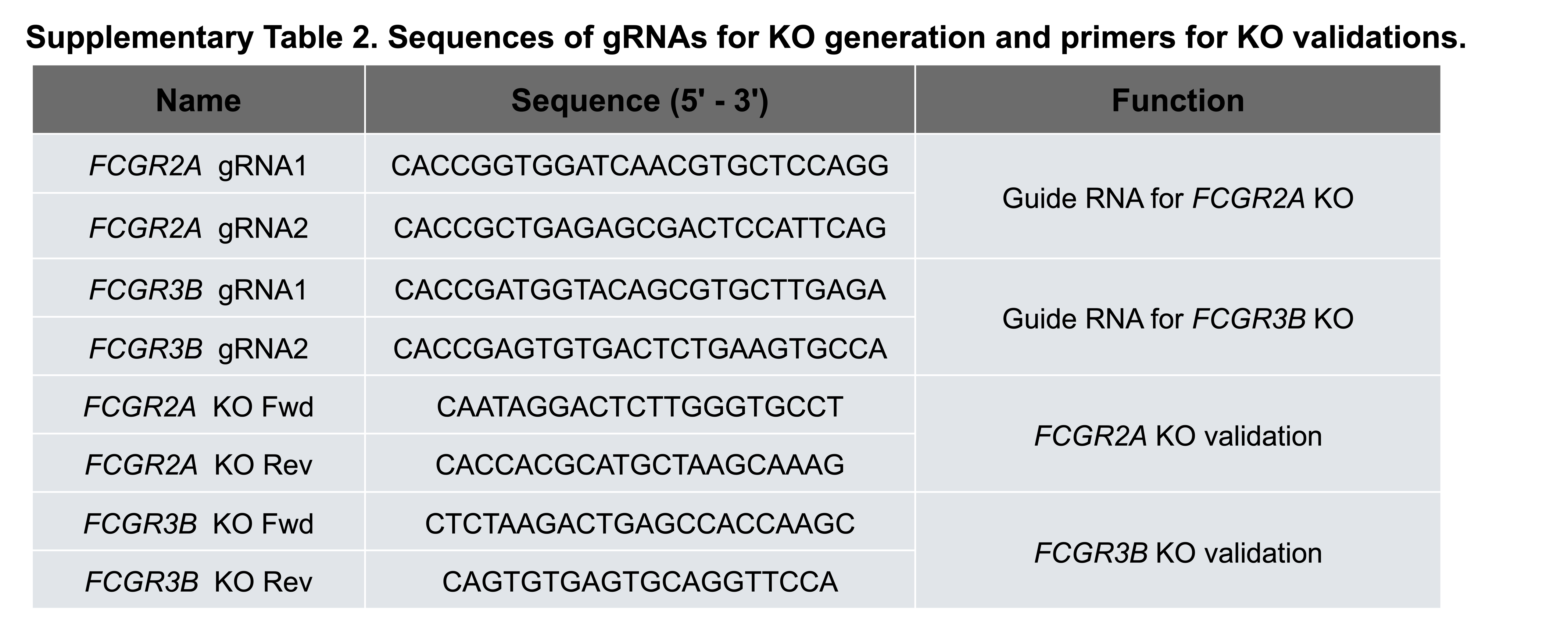

Supplement: Supplementary Table 2 — Sequences of gRNAs for KO generation and primers for KO validations. [file Image9.tiff]

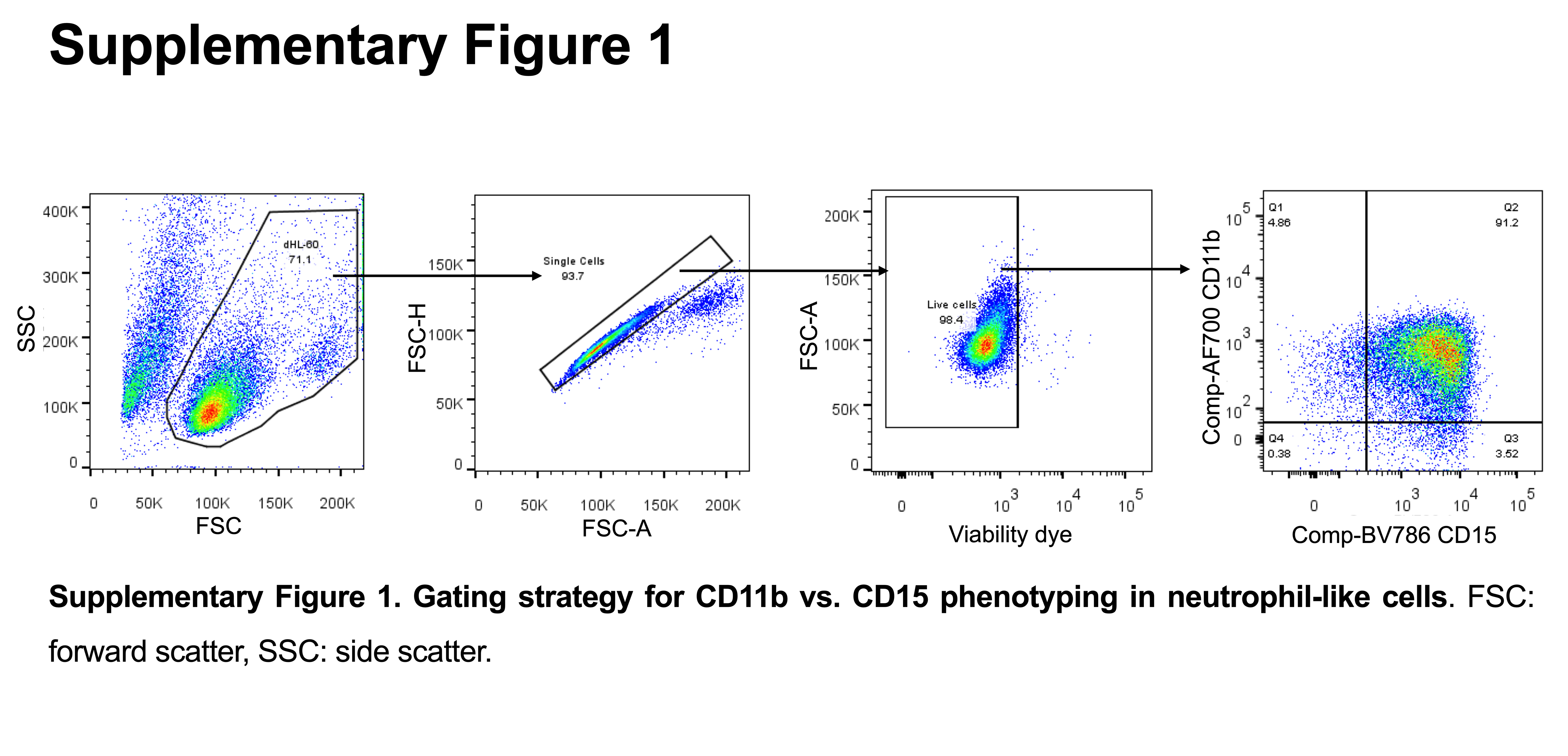

Supplement: Supplementary Figure 1 — Gating strategy for CD11b vs. CD15 phenotyping in neutrophil-like cells. FSC, forward scatter; SSC, side scatter. [file Image1.tiff]

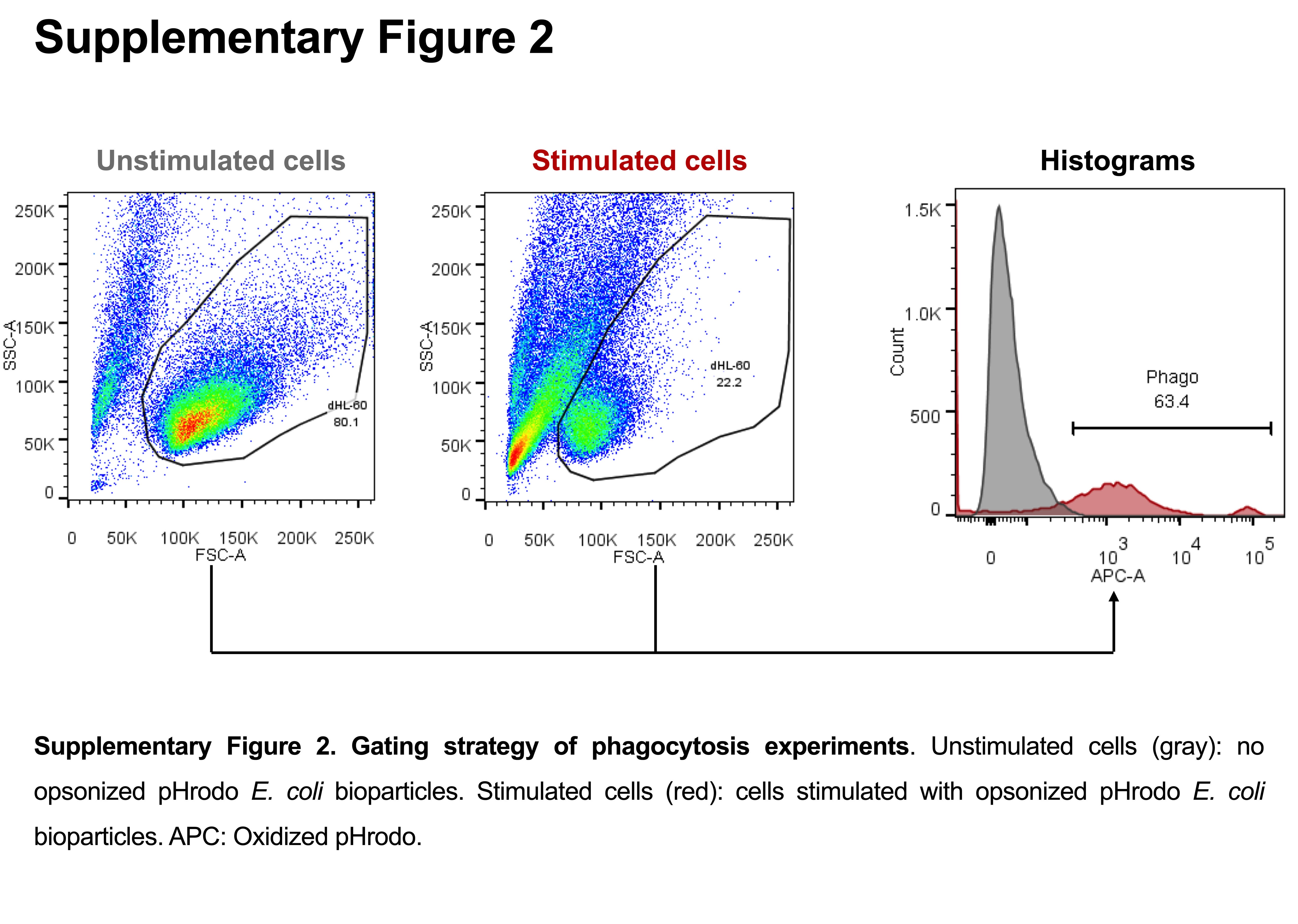

Supplement: Supplementary Figure 2 — Gating strategy of phagocytosis experiments. FSC, forward scatter; SSC, side scatter. Unstimulated cells (gray), no opsonized pHrodo E. coli bioparticles. Stimulated cells (red), cells stimulated with opsonized pHrodo E. coli bioparticles. APC, Oxidized pHrodo. [file Image2.tiff]

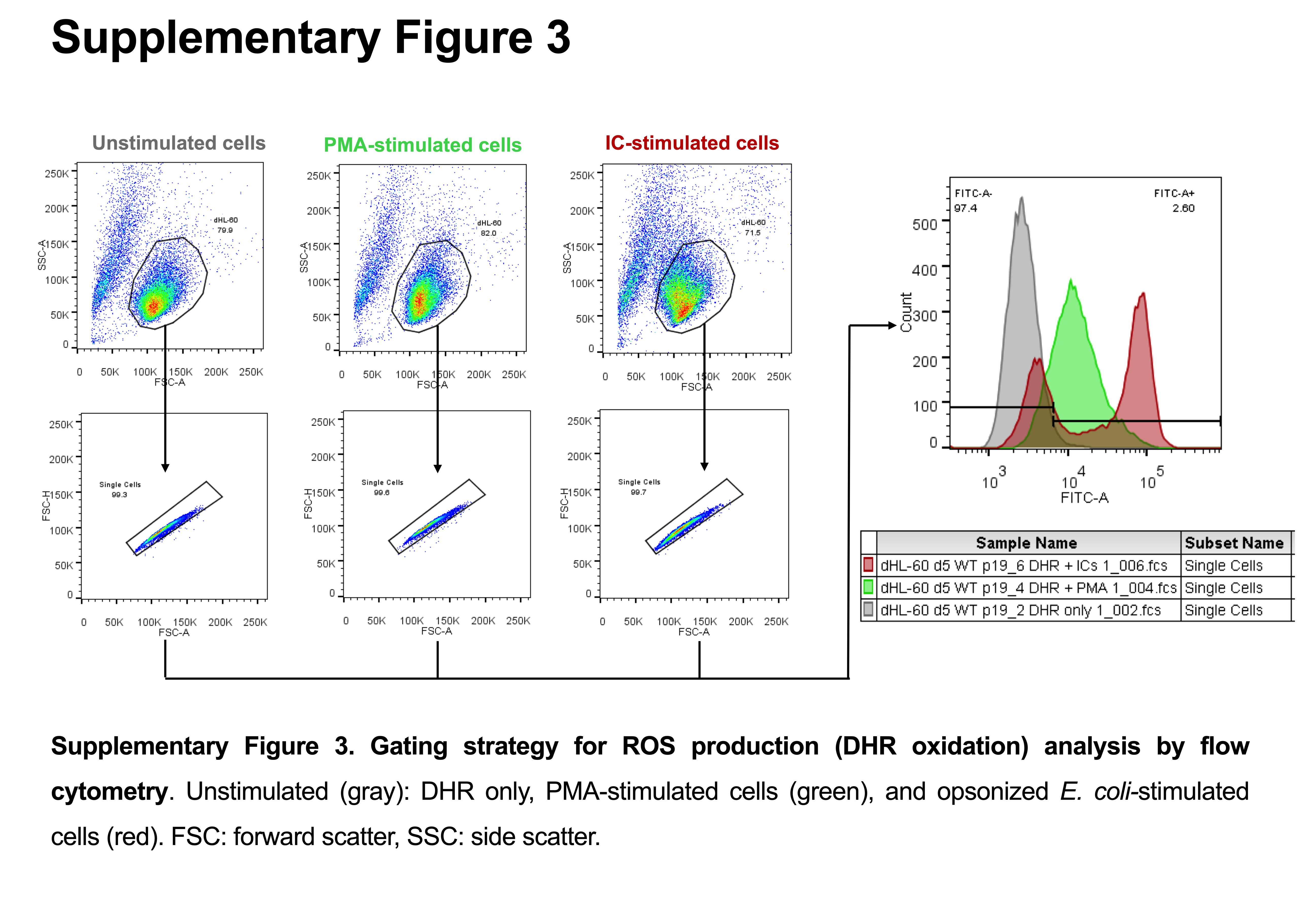

Supplement: Supplementary Figure 3 — Gating strategy for ROS production (DHR oxidation) analysis by flow cytometry. Unstimulated (gray): DHR only, PMA-stimulated cells (100 ng/mL) (green), and opsonized E. coli-stimulated cells (red). FSC, forward scatter; SSC, side scatter. [file Image3.tiff]

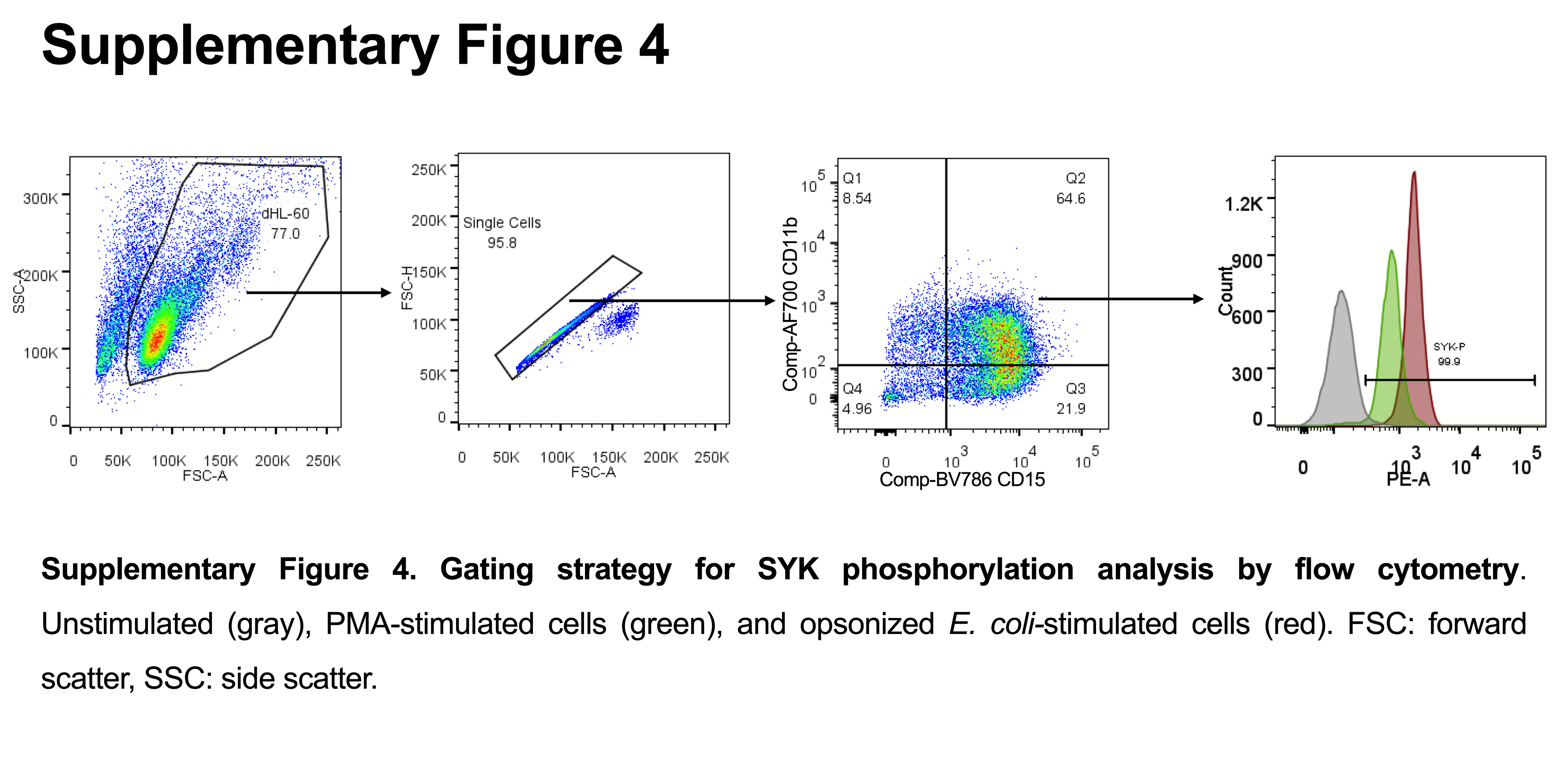

Supplement: Supplementary Figure 4 — Gating strategy for SYK phosphorylation analysis by flow cytometry. Unstimulated (gray), PMA-stimulated cells (green), and opsonized E. coli-stimulated cells (red). FSC, forward scatter; SSC, side scatter. [file Image4.tiff]

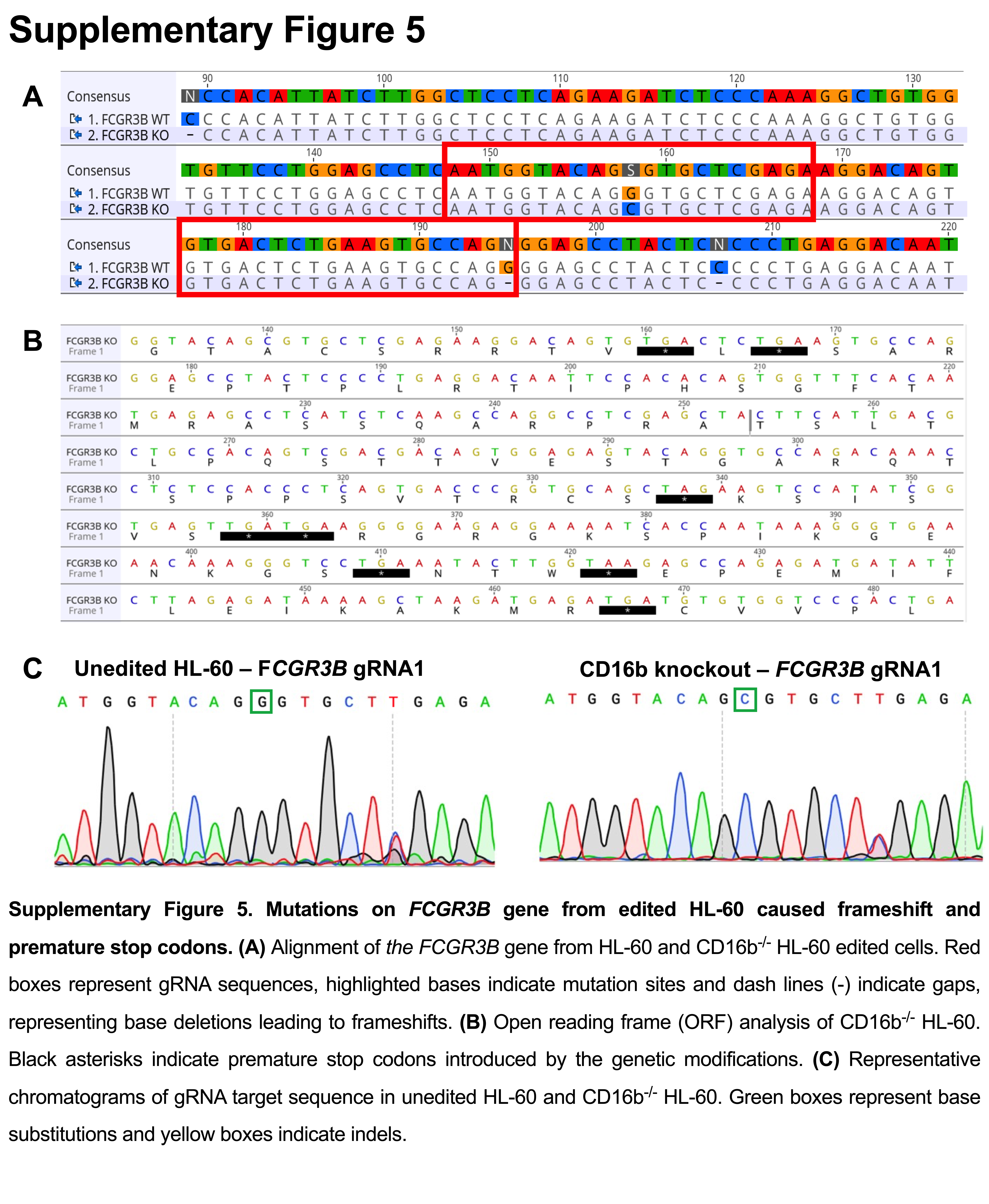

Supplement: Supplementary Figure 5 — Mutations on FCGR3B gene from edited HL-60 caused frameshift and premature stop codons. (A) Alignment of the FCGR3B gene from HL-60 and CD16b-/- HL-60 edited cells. Red boxes represent gRNA sequences, highlighted bases indicate mutation sites and dash lines (-) indicate gaps, representing base deletions leading to frameshifts. (B) Open reading frame (ORF) analysis of CD16b-/- HL-60. Black asterisks indicate premature stop codons introduced by the genetic modifications. (C) Representative chromatograms of gRNA target sequence in unedited HL-60 and CD16b-/- HL-60. Green boxes represent base substitutions and yellow boxes indicate indels. [file Image5.tiff]

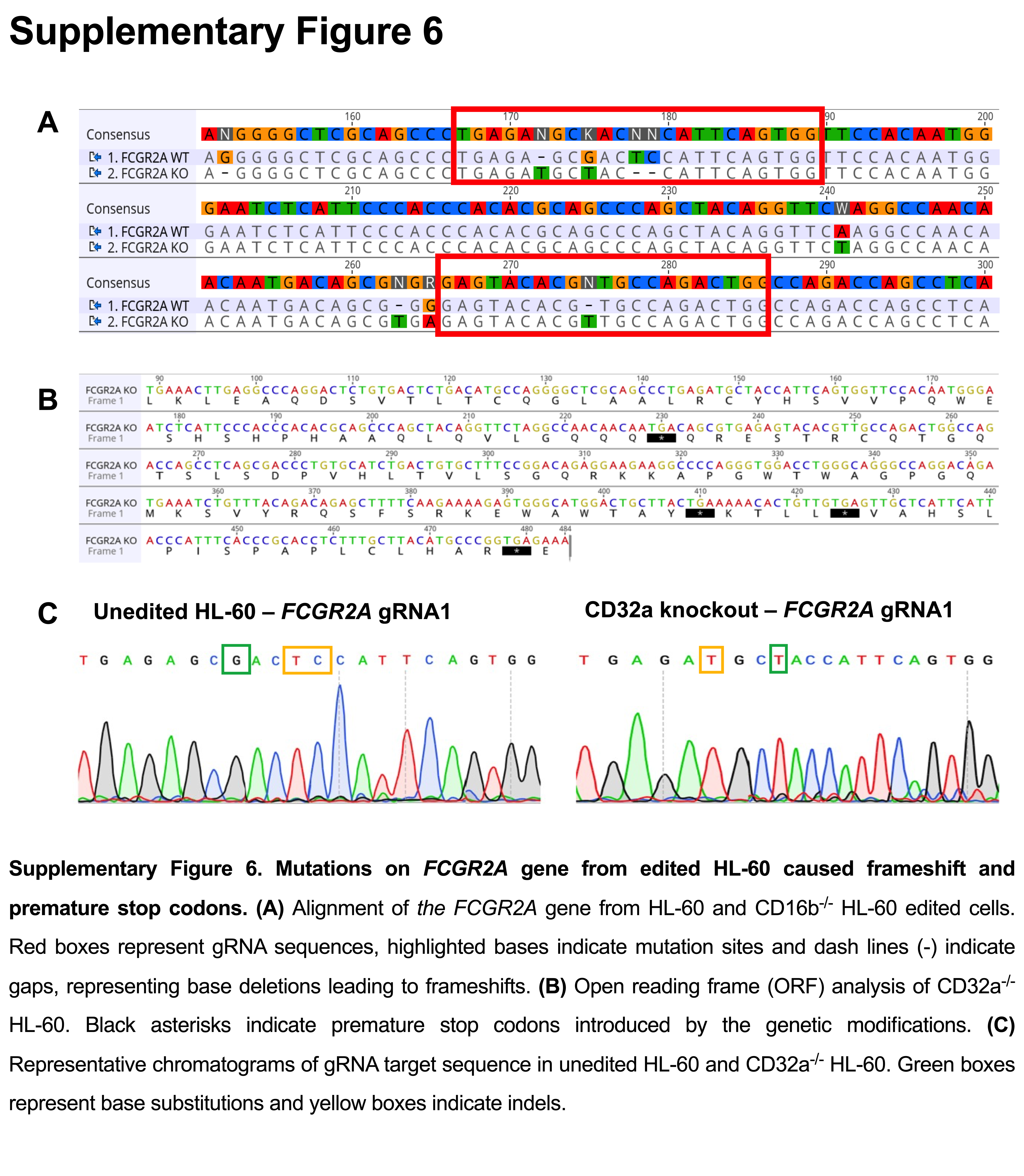

Supplement: Supplementary Figure 6 — Mutations on FCGR2A gene from edited HL-60 caused frameshift and premature stop codons. (A) Alignment of the FCGR2A gene from HL-60 and CD16b-/- HL-60 edited cells. Red boxes represent gRNA sequences, highlighted bases indicate mutation sites and dash lines (-) indicate gaps, representing base deletions leading to frameshifts. (B) Open reading frame (ORF) analysis of CD32a-/- HL-60. Black asterisks indicate premature stop codons introduced by the genetic modifications. (C) Representative chromatograms of gRNA target sequence in unedited HL-60 and CD16b-/- HL-60. Green boxes represent base substitutions and yellow boxes indicate indels. [file Image6.tiff]

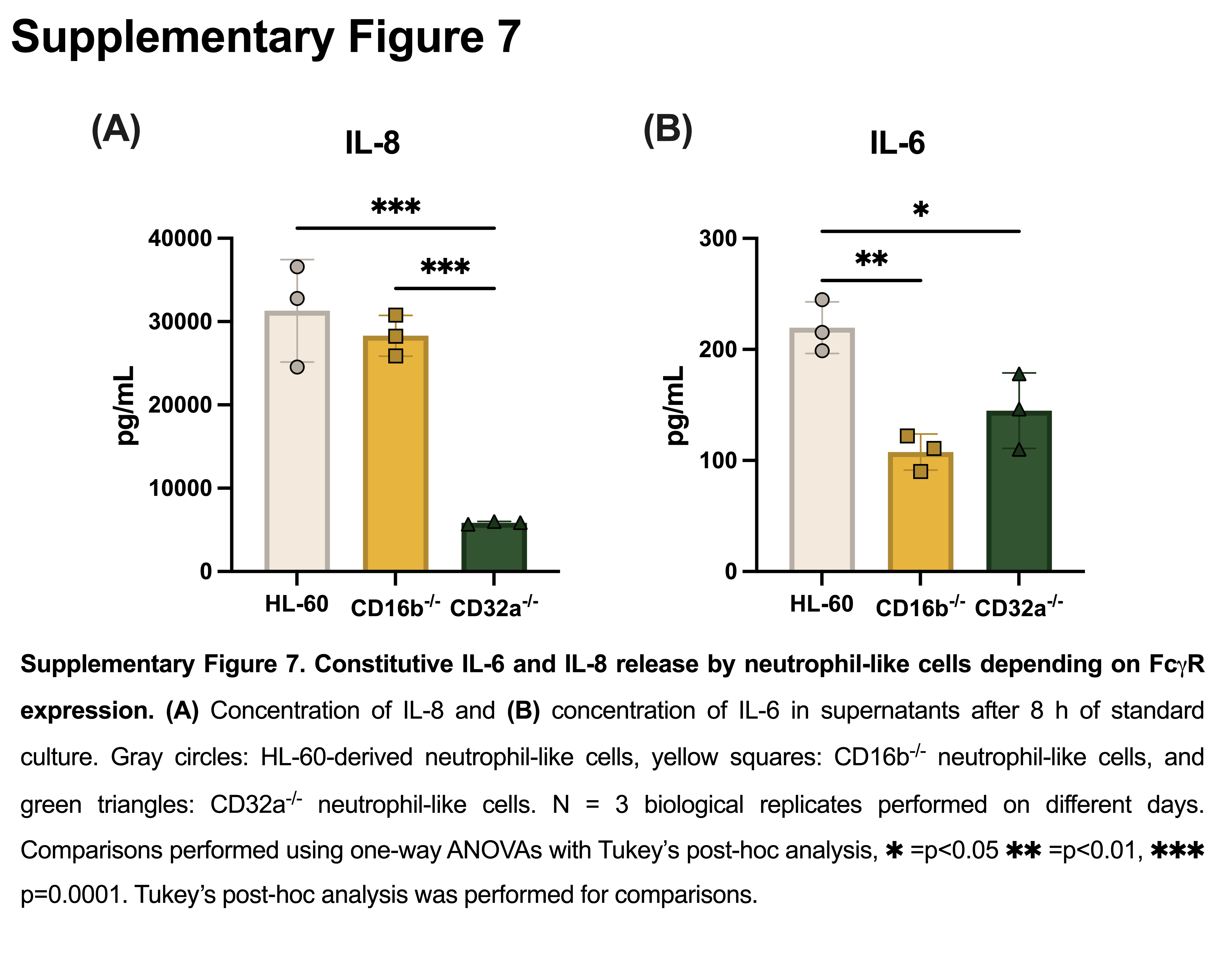

Supplement: Supplementary Figure 7 — Constitutive IL-6 and IL-8 release by neutrophil-like cells depending on FcγR expression. (A) Concentration of IL-8 and (B) concentration of IL-6 in supernatants after 8 h of standard culture. Gray circles: HL-60-derived neutrophil-like cells, yellow squares: CD16b-/- neutrophil-like cells, and green triangles: CD32a-/- neutrophil-like cells. N = 3 biological replicates performed on different days. Comparisons performed using one-way ANOVAs, ✱p<0.05 ✱✱p<0.01, ✱✱✱p=0.0001. [file Image7.tiff]
